# Modulating effect of glutathione (GSH) on 2,4-dichlorophenoxyacetic acid (2,4-D) toxicity

**DOI:** 10.1038/s41598-025-15616-2

**Published:** 2025-08-13

**Authors:** Marzena Matejczyk, Edyta Juszczuk-Kubiak, Paulina Średnicka, Piotr Ofman, Grażyna Łaska, Krzysztof Kurek, Kavindra Kumar Kesari, Ruslan Oblap, Józefa Wiater, Paweł Kondzior, Monika Kalinowska

**Affiliations:** 1 Faculty of Civil Engineering and Environmental Sciences, Department of Chemistry, Biology and Biotechnology, Bialystok University of Technology, Wiejska 45E Str, Bialystok, 15-351 Poland; 2 Department of Biotechnology, Wacław Dąbrowski Institute of Agricultural and Food Biotechnology-State Research Institute, Rakowiecka 36 Str, Prof, Warsaw, 02-532 Poland; 3 Laboratory of Biotechnology and Molecular Engineering, Department of Microbiology, Wacław Dąbrowski Institute of Agricultural and Food Biotechnology-State Research Institute, Rakowiecka 36 Str, Prof, Warsaw, 02-532 Poland; 4 Department of Environmental Engineering Technology, Bialystok University of Technology, Wiejska 45E Str, Bialystok, 15-351 Poland; 5 Department of Agri-Food Engineering and Environmental Management, Bialystok University of Technology, Bialystok, Poland; 6 Department of Gastroenterology and Internal Medicine, Medical University of Bialystok, M. Skłodowskiej-Curie 24 A Str, Bialystok, 15-276 Poland; 7 University Center for Research and Development, Chandigarh University, Mohali, Punjab India; 8 Department of Surgical Medicine with the Laboratory of Medical Genetics, Jan Kochanowski University in Kielce, Collegium Medicum, IX Wieków Kielc 19 A Av, Kielce, 25-317 Poland

**Keywords:** 2,4-dichlorophenoxyacetic acid (2,4-D), Toxicity, Genotoxicity, Microbial biosensor, Estrogenic/androgenic potential, Cancer, Cancer, Environmental sciences, Health care, Risk factors

## Abstract

2,4-dichlorophenoxyacetic acid (2,4-D) is a chlorinated aromatic hydrocarbon herbicide and one of the most widely used herbicides globally. Due to the intensive use of 2,4-D, mainly in agriculture and horticulture, significant amounts of the compound and its metabolites are released into the environment, surface water and soil, posing a serious threat to human health. Scientific studies have shown a positive relationship between 2,4-D exposure and the risk of lymphatic system and prostate cancers. The harmful effects of pesticides and their metabolites on human health can be mitigated by consuming products rich in natural antioxidants, such as glutathione (GSH). This study aimed to elucidate the mechanisms underlying toxicity and genotoxicity of 2,4-D and evaluate the impact of GHS supplementation on mitigating these adverse effects. The toxicity of 2,4-D at the concentrations of 100, 10, 1, 0.1, and 0.01 mg/L was determined by antimicrobial activity against *Enterobacter hormaechei* and *Candida albicans*. In contrast, genotoxicity was determined by the level of induction of the genotoxin-sensitive *recA* promoter in *E. coli* RFM443 *recA:luxCDABE* biosensor strain. Synthesis of reactive oxygen species (ROS) in the *E. coli* strainleads to the oxidative stress response. Moreover, the estrogenic/androgenic effects of 2,4-D were evaluated by yeast estrogen (YES) and androgen (YAS) screen assay using the genetically modified *S. cerevisiae* strain at various concentrations (100, 10, 1, 0.1, 0.01, 0.001, 0.0001, and 0.00001 mg/L). Finally, the effect of 2,4-D mixtures with GSH at a concentration of 1 mg/L on mitigating its toxic and genotoxic activity was investigated. In the mixtures, the concentration of GSH was lower than the physiological concentration in the cells, and it was selected experimentally to obtain satisfactory results. The experiment was conducted in three independent series, with at least three repetitions of each result (*n* = 3). Results showed that 2,4-D in the range of applied concentrations exerted a toxic effect on *E. homaechei* and *C. albicans* strains and a genotoxic effect in *E. coli recA:luxCDABE* biosensor strain. Analysis of ROS synthesis values in the *E. coli* strain showed an increase in this parameter following exposure to the tested 2,4-D concentrations. In the YES and YAS bioassays performed for 2,4-D, we did not detect the ability to stimulate the estrogen/androgen receptor. In mixtures of 2,4-D, a significant (*p* = 0.05) reduction effect on the toxicity, above 7% for *E. hormaechei* and above 16% for *C. albicans* and genotoxicity, by more than 44% of the herbicide was detected after the addition of glutathione This indicating that GSH taken up with food or in the form of supplements can mitigate the adverse effects of 2,4-D in living cells and protect cells from cancer induction. The results confirmed the validity of the hypothesis that oxidative stress induction is a molecular mechanism of 2,4-D toxicity and genotoxicity. Given the significant environmental and food pollution from pesticides and the link between human exposure and cancer induction, proper dietary choice and consumption of foods rich in glutathione are essential in cancer prevention.

 Cancer is one of the leading causes of premature death in both men and women in the human population worldwide. According to the World Health Organization (WHO) cancer agency and the International Agency for Research on Cancer (IARC) statistical publications in 2022, there were an estimated 20 million new cancer cases and 9.7 million deaths. From the data collected, it was detected that the most common cancers included lung, breast and colorectal cancers^1,2^. Statistics from EU countries have shown that in 2021, cancer was the second leading cause of death in the EU, with 1.1 million deaths, which equated to 21.6% of the total number of deaths in the EU^3^.

Scientific studies have shown a positive relationship between pesticide exposure and cancer risk^4,5^. The main sources of transport of pesticides into the human body are food and water. In EU countries, among the primary documents overseeing the safety of food and feed of plant and animal origin regarding their possible pesticide contamination is Regulation (EC) No 396/2005 of the European Parliament and of the Council of 23 February 2005. This document clearly defines the maximum residue levels for pesticides. In contrast, the pesticide content of drinking water is regulated by the Drinking Water Directive (EU) 2020/2184, which allows a maximum pesticide content up to a limit of 0.1 µg/L^6^.

European Food Safety Authority’s (EFSA) latest report on pesticide residues in food in the European Union is worrying. In 2021, a total of 87,863 food samples from 12 food products such as aubergines, bananas, broccoli, cultivated mushrooms, grapefruit, melons, sweet peppers, table grapes, extra virgin olive oil, wheat, beef fat and hen eggs were tested for pesticides in EU countries. About 40% of the tested samples contained one or more residues at concentrations below or equal to the permitted levels. In contrast, more than 2% contained residues above permitted levels^7^. European surface and groundwater, often used as drinking water sources, also contain significant pesticides. According to the European Environment Agency (EEA) report, 25% of all reported monitoring sites in European surface waters in 2019 were found to exceed the pesticide limit of 0.1 µg /L. The main pesticides detected in water above the permitted limit included insecticide imidacloprid, herbicide metolachlor, atrazine and its metabolites and bentazone^8^. Pesticides are also present in Polish waters. A study by Kruć-Fijałkowska et al. (2022) detected significant concentrations of pesticides in the Warta River, ranging from 0.031 to 0.472 µg/L. The most frequently detected pesticides were isoproturon, nicosulfuron, imidacloprid, terbuthylazine, chlorotoluron, S-metalachlor, and prometryn^9^. In India, pesticide content in Ganga river waters reached values as high as 10.402 µg/L^10^.

2,4-dichlorophenoxyacetic acid (2,4-D) is a chlorinated aromatic hydrocarbon herbicide (CAH) (Fig. 1) that is one of the most applied worldwide, introduced on the market in the 1940s to control broad-leaved weeds such as wheat, soybean, corn, and other crops^11,12^. 2,4-D is a synthetic auxinic plant growth regulator that mimics the activity of natural plant auxins at lower concentrations, promoting cell division, differentiation, and elongation^13^. However, at higher concentrations, it significantly disrupts metabolic processes in plant cells, causing growth inhibition, abnormalities, chloroplast damage, and ultimately plant death^13,14^. 2,4-D owes its popularity in application to its physical and chemical properties such as selectivity, broad-spectrum application and low cost. In addition, a critical feature of this herbicide is its relatively good water solubility, which ensures good penetration into plant tissues and the root system, increasing the herbicide’s effectiveness. 2,4-D as an active ingredient is included in some 1,500 different herbicides, and the exemplary consumption of 2,4-D in the form of 2,4-D butyl ester in China reaches a value of 5,000–8,000 tonnes annually^11,12^.

**Fig. 1:**
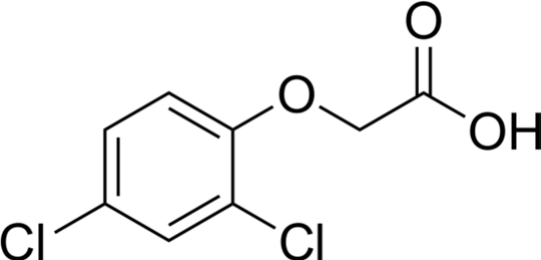
Chemical structure of 2,4-dichlorophenoxyacetic acid (2,4-D).

The main transformation products of 2,4-D formed during environmental degradation in oxidation, reduction, photolysis, and hydrolysis reactions are 1,2,4-benzenetriol, 4-chlorophenol and 2,4-dichlorophenol^12^. A significant proportion of them show toxicity to living organisms. Due to the continuous increase in the consumption of 2,4-D, its residues are being detected in wastewater, surface and groundwater as potential drinking water intakes, in soils, and drinking water and process water used in the agri-food industry^15–17^. In 2021, more than 62,000 tonnes of 2,4-D were sold in Brazil. By contrast, this herbicide was found in approximately 600 agricultural and residential products in the United States. In Brazil, during the 2015–2016 monitoring of surface freshwater, 2,4-D was detected in 20% of the samples analysed at concentrations well above the limits set by the aquatic life protection criteria and reaching values even to 366 µg/L^18^. In Europe, 2,4-D was detected in drinking and surface water in Spain at a concentration of 207 ng/L. Even higher concentrations of 2,4-D up to 12 µg/L were detected in urban surface waters and sediments within the USA^19^. The highest recorded EPA and WHO concentrations of 2,4-D in drinking water are 0.07 mg/L and 30 µg/L, respectively^20^. 2,4-D is a persistent xenobiotic with an environmental half-life in water of up to 300 days^19^. Moreover, this herbicide has a low sorption potential into soil particles, resulting in the frequent occurrence of 2,4-D as a pollutant within Europe in lakes and rivers in concentrations, exceeding the European Union threshold (0.1 µg/L) for drinking water sources^6,11^. Earlier studies demonstrated the toxic effect of 2,4-D on aquatic biota, such as algae, chironomids, and fish^21,22^. In recent years, hepatotoxicity of 2,4-D, including liver cellular and tissue damage, has been confirmed using rodent and fish models^19^. In humans, exposure to 2,4-D has been linked to heightened risks of cancer, neurological diseases, reproductive issues, and immunotoxicity^20^.

2,4-D is incorporated into the food chain and can be detected in animal tissues, plants, and animal products such as eggs and milk. As the last link in the food chain, humans consume both contaminated foods of plant and animal origin. Once 2,4-D enters the human body, it is metabolised and excreted mainly in the urine, either unchanged or as biologically active and often toxic metabolites^22,23^. Scientific studies have shown that 2,4-D can induce oxidative stress in human cells. Moreover, this herbicide has been shown to strongly affect the human immune system by inducing immunosuppression. Both oxidative stress and immune response disorders contribute to the predisposition for cancer development in the human body^24,25^. Scientific studies have shown the existence of an increased risk of Non-Hodgkin’s lymphoma (NHL, lymphatic system cancers) and prostate cancer, especially in people directly exposed to 2,4-D like farmers or professional applicators^26–28^. Based on scientific results, the IARC classified 2,4-D as “possibly carcinogenic to humans” (Group 2B)^2^.

The harmful effects of pesticides and their metabolites on the human body can be mitigated by consuming products rich in natural antioxidants, such as glutathione (GSH). Glutathione is an organic chemical compound. It is a tripeptide consisting of three amino acids (glycine, L-cysteine and L-glutathione acid)^29^. The antioxidant properties of the glutathione molecule are related to the presence of a thiol group (-SH) in cysteine. It occurs in plant and animal organisms in two forms: reduced (GSH) and oxidized (GSSG). GSH is abundant in cruciferous vegetables, citrus fruits and animal products^30–34^. To support glutathione synthesis, which occurs primarily in the liver, it is crucial to include in the diet foods high in vitamin C, selenium, and amino acids such as cysteine and methionine^30,32,35,36^.

Glutathione is a natural antioxidant synthesised in human cells^30,35,36^. 2,4-D, on the other hand, is a commonly used pesticide that enters the human body via inhalation (soil spraying), ingestion (along with food and water contaminated with this pesticide) and through the skin (direct contact)^15–20^. Hence, there is a high probability of interaction in human cells between 2,4-D and GSH. Investigating these interactions is a unique contribution to the knowledge of GSH activity in 2,4-D-treated cells.

Given the above, the objective of this study was to assess the toxicity and genotoxicity of 2,4-D and to determine whether glutathione (GSH), a key component of the antioxidant system, can mitigate the toxic and genotoxic effects of 2,4-D when present in mixtures with GSH. The toxicity potential was determined by antimicrobial activity against *Enterobacter hormaechei* LBM ATCC-700323 and *Candida albicans* ATCC-10231. Subsequently, the genotoxicity of 2,4-D was estimated by applying *Escherichia coli* RFM443 *recA:luxCDABE* biosensor strain with a genotoxin-sensitive *recA* promoter. To investigate oxidative stress as a potential mechanism of toxicity, we measured the levels of reactive oxygen species (ROS) in the *E. coli* ATCC-25922 strain. *E. coli* and *C. albicans* are part of the natural microbiota of the human body and are commonly found in environmental matrices such as sewage, waters and soils^37,38^. Additionally, to evaluate the binding affinity of 2,4-D to estrogen and androgen receptors, we performed yeast estrogen screen (YES) and yeast androgen screen (YAS) assays using a genetically modified strain of *Saccharomyces cerevisiae.*

## Results

### 2,4-D, Nalidixic acid (NA) and their mixtures with GSH antimicrobial activity

**Fig. 2:**
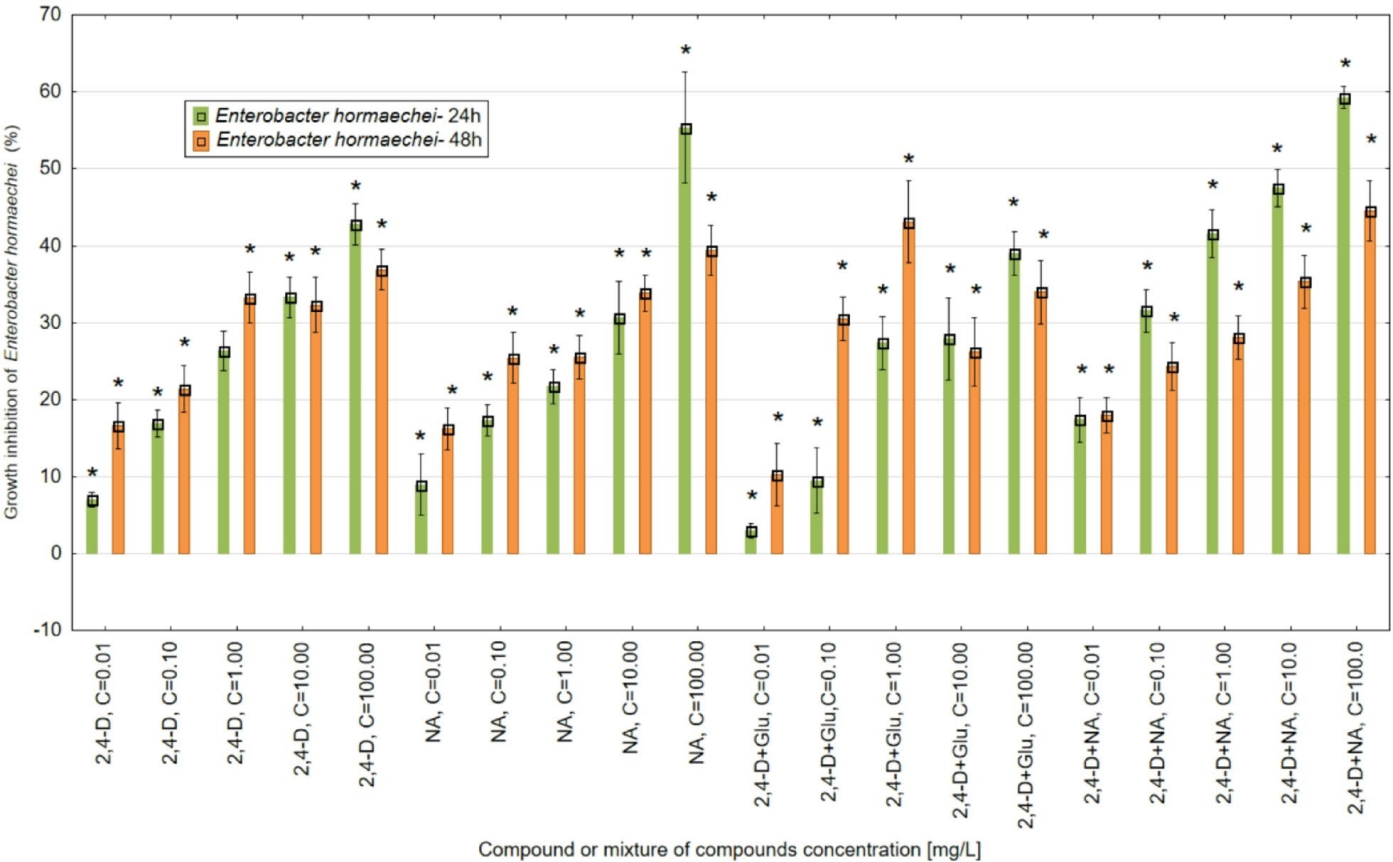
The antimicrobial activity of 2,4-D, NA (applied at the concentrations 0.01, 0.1, 1, 10 and 100 mg/L) and 2,4-D mixtures with NA and glutathione (Glu) used at the concentration of 1 mg/L against *E. hormaechei* LBM ATCC-700323 after 24 and 48 h incubation with the tested chemicals. The results obtained for mixtures with GSH were standardised against GSH. Note: *- statistically significant differences at *p* = 0.05 between the control sample (100%). The results are presented as mean ± SD (standard deviation). Error bars indicate the SD of the mean of three independent replicates (*n* = 3).

**Fig. 3:**
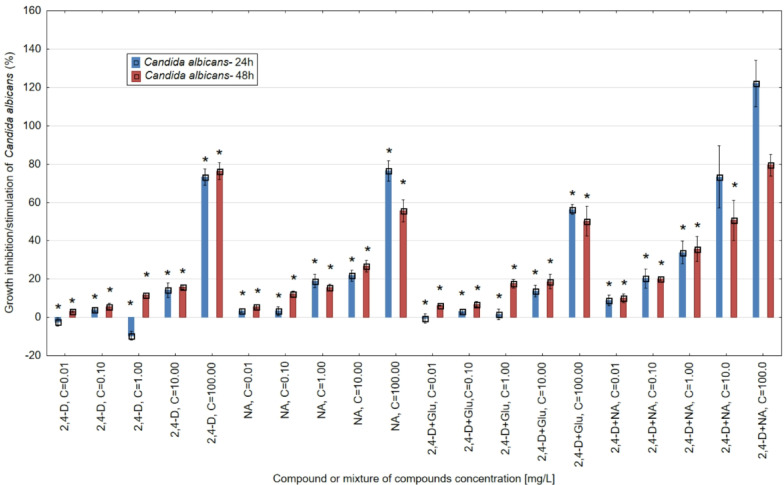
The antimicrobial activity of 2,4-D, NA (applied at the concentrations 0.01, 0.1, 1, 10 and 100 mg/L) and 2,4-D mixtures with NA and glutathione (Glu) used at the concentration of 1 mg/L against *C. albicans* ATCC-10231 after 24 and 48 h incubation with tested chemicals. The results obtained for mixtures with GSH were standardised against GSH. Note: *- statistically significant differences at *p* = 0.05 between the control samples. The results are presented as mean ± SD (standard deviation). Error bars indicate the SD of the mean of three independent replicates (*n* = 3).

The results showed that GSH, in the range of concentrations tested, modulated the toxicity of 2,4-D towards *E. hormaechei* LBM ATCC-700323 and *C. albicans* ATCC-10231 strains after 24 and 48 h of the microorganism’s incubation with pesticide and its mixtures with GSH (Figs. 2 and 3).

For *E. hormaechei*, after a 24-hour incubation with various concentrations of 2,4-D, the strongest inhibitory effects on bacterial culture growth were observed at the highest concentrations of 100, 10, and 1 mg/L, reducing growth by over 40%, 30%, and 25%, respectively, compared to the control. Lower concentrations of 2,4-D (0.1 and 0.01 mg/L) exhibited less antimicrobial activity, inhibiting culture growth by 16% and 7%, respectively. Compared to analogous concentrations of 2,4-D, the parallel test standard NA differed by more than 13% higher antimicrobial activity against *E. hormaechei* at the highest concentration of 100 mg/L after 24 h. At the other NA concentrations (10, 1, 0.1 and 0.01 mg/L), its toxic activity against the *E. hormaechei* strain was comparable to 2,4-D tested at the same concentrations. In mixtures of 2,4-D with glutathione (GSH), adding GSH at a concentration of 1 mg/L reduced the toxicity of 2,4-D applied at 100 mg/L by more than 3%. At a concentration of 10 mg/L of pesticide, GSH reduced toxicity by approximately 6%. Furthermore, at a concentration of 0.1 mg/L of 2,4-D, the toxicity was reduced by more than 7%, while at the lowest concentration of 0.01 mg/L, the reduction in toxicity exceeded 4%. The addition of NA standard at 1 mg/L in mixtures with 2,4-D tested at concentrations of 100,10,1,0,1 and 0.01 mg/L increased the toxicity of the pesticide to the *E. hormaechei* strain tested after 24 h. The highest antimicrobial activity enhancement values of about 17% for 2,4-D were detected when GSH at a concentration of 100 mg/L was added to the pesticide, compared to 2,4-D applied alone. For the remaining concentrations of 2,4-D (10.1,0.1 and 0.01 mg/L), the addition of GSH at 1 mg/L elevated the toxicity of the pesticide from more than 10% (for a concentration of 0.01 mg/L 2,4-D) to more than 15% for a concentration of 1 mg/L 2,4-D. Extending the incubation time of *E. hormaechei* cultures with 2,4-D and NA to 48 h enhanced their toxic effects, mainly at the lower concentrations tested (1, 0.1 and 0.01 mg/L) of both chemicals. For 2,4-D, the highest level of toxicity stimulation after 48 compared to 24 h of more than 9% was obtained at a concentration of 0.01 mg/L. In the case of NA over 8%, the difference in toxicity values between the analyzed incubation times (24 h and 48 h) was observed at a 0.1 mg/L concentration. In mixtures of 2,4-D (1, 0.1 mg/L) with GSH, extending the incubation time of the bacterial strain to 48 h causes an increase in the toxicity of the mixtures by about 10% compared to 2,4-D used alone. In the case of higher concentrations of 2,4-D and NA (100, 10 mg/L) and compared to 24 h, a decrease of about 6% (for 2,4-D) and 15% (for NA) in the toxicity of both tested chemical compounds was detected. In comparison to 24 h and at most of the tested 2,4-D concentrations, a decrease in the toxicity of 2,4-D with NA mixtures was also observed.

*C. albicans* ATCC-10231 applied at a concentration of 100 mg/L showed the most significant sensitivity to 2,4-D after 24 h and 48 h of incubation, respectively, up to 73.13% (24 h) and 76.40% (48 h) of growth inhibition in comparison to the control sample (Fig. 3).

At concentrations of 1 and 0.1 mg/L 24 h, a slight stimulation of the growth of *C. albicans* cultures was detected by approximately 10% and 2.5%, respectively. The 24 h exposure of fungi cells to NA induced a toxic effect on culture growth inhibition of above 3% (0.1 and 0.01 mg/L) to above 76% (100 mg/L). For 2,4-D, extending the incubation of *C. albicans* to 48 h slightly increased its toxicity at all the concentrations tested. In contrast, in the case of the NA standard used, at most of the concentrations tested, extending its duration of action to 48 h induced a reduction in toxic potential against the fungus culture. After 24 h, in mixtures, GSH added to 2,4-D at a concentration of 100 mg/L reduces by 16.77% the toxicity of the pesticide. Increasing from 24 h to 48 h the duration of action of mixtures of 2,4-D with GHS on fungal cells at the highest pesticide concentration (100 mg/L) reduces the mixture’s toxicity by more than 6%. At the other concentrations (10, 1, 0.1 and 0.01 mg/L) of the pesticide, there is an increase in toxicity, even to about 16% in the mixture with 1 mg/L 2,4-D. After 48 h, GSH in mixtures with 2,4-D only at the highest tested concentration of 2,4-D (100 mg/L) reduces the toxicity of the pesticide by more than 26% compared to 2,4-D used alone. For the other concentrations of the pesticide in mixtures with GSH, the toxicity of 2,4-D increased from more than 1% (0.1 mg/L 2,4-D) to more than 6% (1 mg/L 2,4-D) was observed. A 48-h exposure of *C. albicans* compared to 24 h reduced the toxicity of 2,4-D mixtures with NA, especially at the highest 2,4-D concentrations of 100 and 10 mg/L by more than 40% and more than 20%, respectively. In these mixtures, both after 24 h and 48 h, an enhancement of the toxic potential against the fungus culture was detected compared to the toxicity of the individual components of the mixtures (2,4-D and NA). 24 h and 48 h exposure of *E. hormaechei* and *C. albicans* cultures to GSH alone at a concentration of 1 mg/L induced growth stimulation of cultures of both microorganisms by more than 9% (24 h *E. hormaechei*) and more than 5% (48 h *E. hormaechei*) by more than 10% (24 h *C. albicans*) and more than 5% (48 h *C.albicans*), respectively.

From the research carried out on the antimicrobial activity of 2,4-D, NA and its mixtures with GSH using the *Enetrobacter hormaechei* and *Candida albicans* strains, it was observed that the activity of the essential compounds and their mixtures contribute to the occurrence of statistically significant differences in the values of this indicator after 24 h and does not progress with an increase in exposure time to 48 h. This observation is driven by the results of the Tukey test for control samples, where statistically significant differences (*p* = 0.05) were observed between the control samples and the values observed after 24 h of incubation. Similarly, for the 48 h incubation, statistically significant differences were observed between the values obtained in the control samples. However, statistically significant differences in the microbial activity of individual compounds and their mixtures were not observed between samples after incubation times of 24 and 48 h.

### The genotoxicity determination of 2,4-D, NA and their mixtures with GSH

**Fig. 4:**
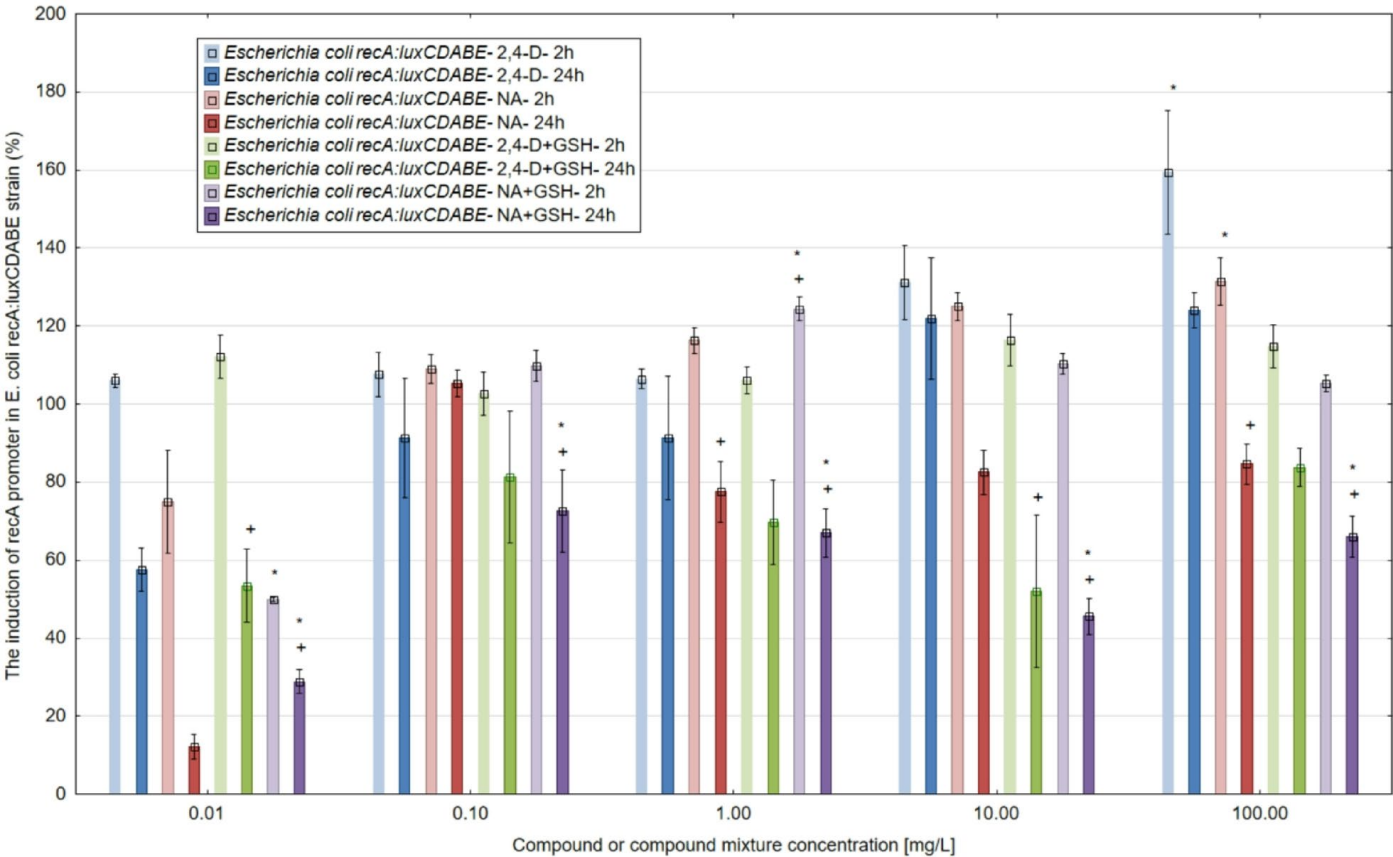
The genotoxic effect of 2,4-D, NA (applied at the concentrations 0.01, 0.1, 1, 10 and 100 mg/L) and their mixtures with glutathione (GSH) used at the concentration of 1 mg/L in *Escherichia coli* biosensor strain RFM443 *recA:luxCDABE* compared to time 0 and after 2 and 24 h incubation with tested chemicals. The results obtained for mixtures with GSH were standardised against GSH. Note: *- statistically significant differences at α = 0.05 standardized with time 0; + statistically significant differences at *p* = 0.05 between 2 and 24 h. The results are presented as mean ± SD (standard deviation). Error bars indicate the SD of the mean of three independent replicates (*n* = 3).

A two-hour incubation of *E. coli* RFM443 *recA:luxCDABE* with 2,4-D and NA at the tested concentrations of 100, 10, 1, 0.1 and 0.01 mg/L caused an induction of the *recA* promoter and an increase in luminescence, indicating a genotoxic effect of the tested chemicals (Fig. 4). For 2,4-D, the most potent genotoxic effect against bacteria was detected at the highest tested concentrations of 100 and 10 mg/L and was almost 160% and more than 130%, respectively, compared to the control (100%). In contrast, NA used at analogous concentrations induced the *recA* promoter in the *E. coli* strain to values of over 130% (100 mg/L NA) and 120% (10 mg/L NA).

In mixtures of 2,4-D and NA with GSH after 2 h, the addition of GSH at a concentration of 1 mg/L significantly reduced the genotoxic effects of both chemicals. In mixtures with 2,4-D, glutathione reduced the genotoxicity of the herbicide most intensively by more than 44% and 14% at concentrations of 100 and 10 mg/L, respectively. In mixtures of 2,4-D and NA with GSH after 2 h, the addition of GSH at a concentration of 1 mg/L significantly reduced the genotoxic effects of both chemicals. In mixtures with 2,4-D, glutathione reduced the genotoxicity of the herbicide most intensively by more than 44% and 14% at concentrations of 100 and 10 mg/L, respectively. In contrast, in mixtures of GSH with NA, the genotoxicity-reducing effect of glutathione was significantly less intense by more than 18% (100 mg/L NA) compared to mixtures with 2,4-D. In mixtures, glutathione reduced the genotoxicity of 2,4-D more strongly than the genotoxicity-reducing effect in mixtures with NA.

Prolonged incubation of *E. coli* strain RFM443 *recA:luxCDABE* with 2,4-D, NA and their mixtures with GSH up to 24 h resulted in a significant decrease, especially for NA and the mixtures, in genotoxicity. The genotoxicity-reducing effect of adding glutathione to the mixtures was also not detected.

**Fig. 5:**
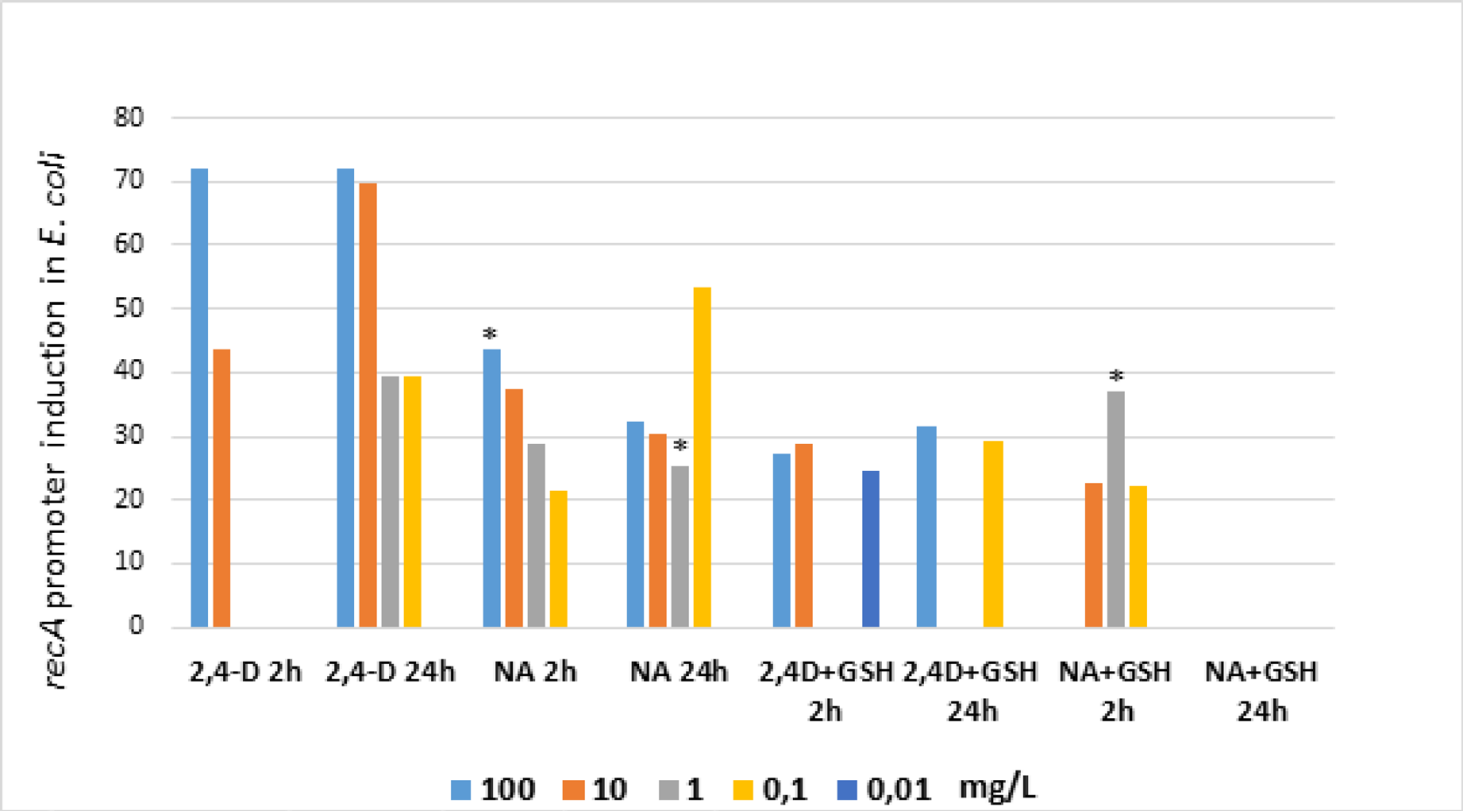
The genotoxicity results normalized with the control sample. The graph shows the results of ≥ 20% induction compared to the control sample Note: *- statistically significant differences at *p* = 0.05 compared to the control sample. Note: *- statistically significant differences at *p* = 0.05 standardized with control sample.

Studies of the *recA* promoter induction in *Escherichia coli* strain RFM443 *recA:luxCDABE* show that, despite a toxic effect in most of the samples observed, statistically significant differences generally occur at incubation times of 24 h. This phenomenon was observed for NA, a mixture of 2,4-D and GSH and a mixture of NA and GSH at concentrations greater than or equal to 1.00 mg/L. In Fig. 5, the genotoxicity results were shown after normalization with the control sample.

In the presented toxicity and genotoxicity studies, we detected GSH’s paradoxical effects (protection vs. toxicity enhancement) - in mixtures containing 2,4-D at a concentration of 0.01 mg/L with GSH, an increase in 2,4-D toxicity (by approximately 6%) was observed. A similar GSH’s paradoxical effect was noticed in a mixture of GSH with NA applied at a concentration of 1 mg/L, with enhancement of more than 8% in the genotoxicity of NA compared to NA applied alone. The molecular mechanism of this phenomenon is described in more detail in the “Discussion” section of this article.

### Yeast Estrogen screen (YES) and androgen screen (YAS) bioassays

**Fig. 6:**
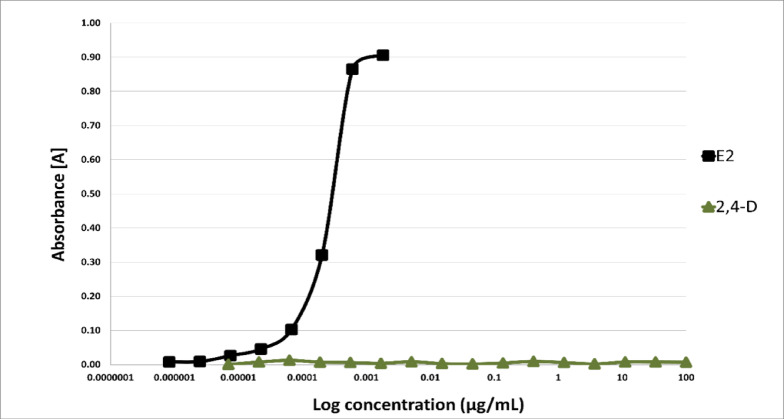
ER dose-response curves for 2,4-dichlorophenoxyacetic acid (2,4-D) and 17β-estradiol (E2) as positive control (*n* = 6). The activity is measured through an estrogenic (YES) assay and represented as EC_20_ (M), (*n* = 3).

**Fig. 7:**
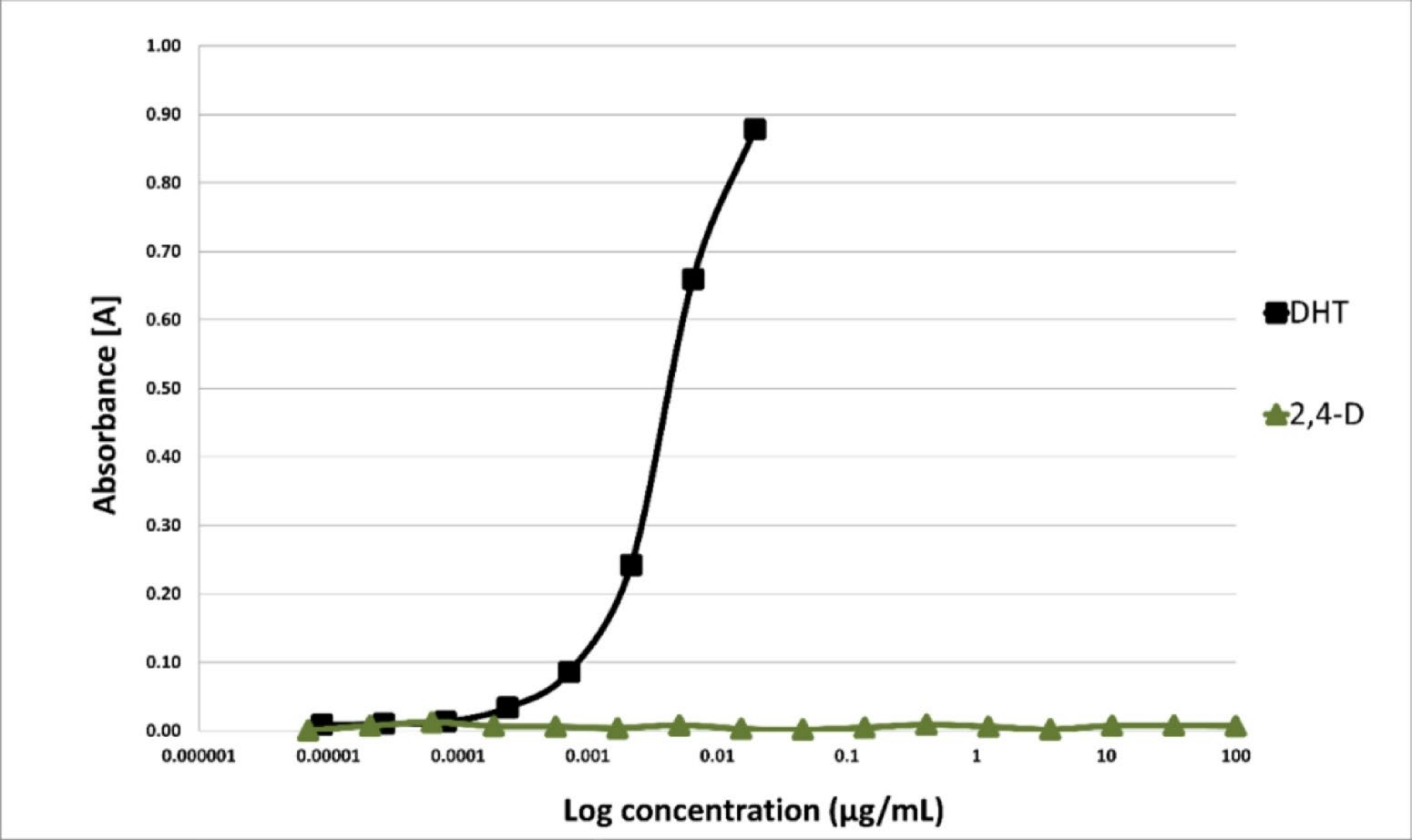
AR dose-response curves for 2,4-dichlorophenoxyacetic acid (2,4-D) and dihydrotestosterone (DHT) as positive control (*n* = 6). The activity is measured through an androgenic (YAS) assay and represented as EC_20_ (M) (*n* = 3).

The potential endocrine-disrupting activity of 2,4-D was assessed using the in vitro YES and YAS bioassays following 48 h of exposure. At the tested chemical concentrations, ranging from 100 to 7.0 × 10^−6^ µg/mL, no inhibition of yeast cell growth was observed, confirming that the obtained results were not affected by changes in yeast growth or chemical toxicity. The results showed that 2,4-D did not exhibit any agonist activity toward human estrogenic (hERα) and androgenic (hAR) receptors across the entire range of tested concentrations (Figs. 6 and 7).

## Oxidative stress determination

### ROS synthesis

**Fig. 8:**
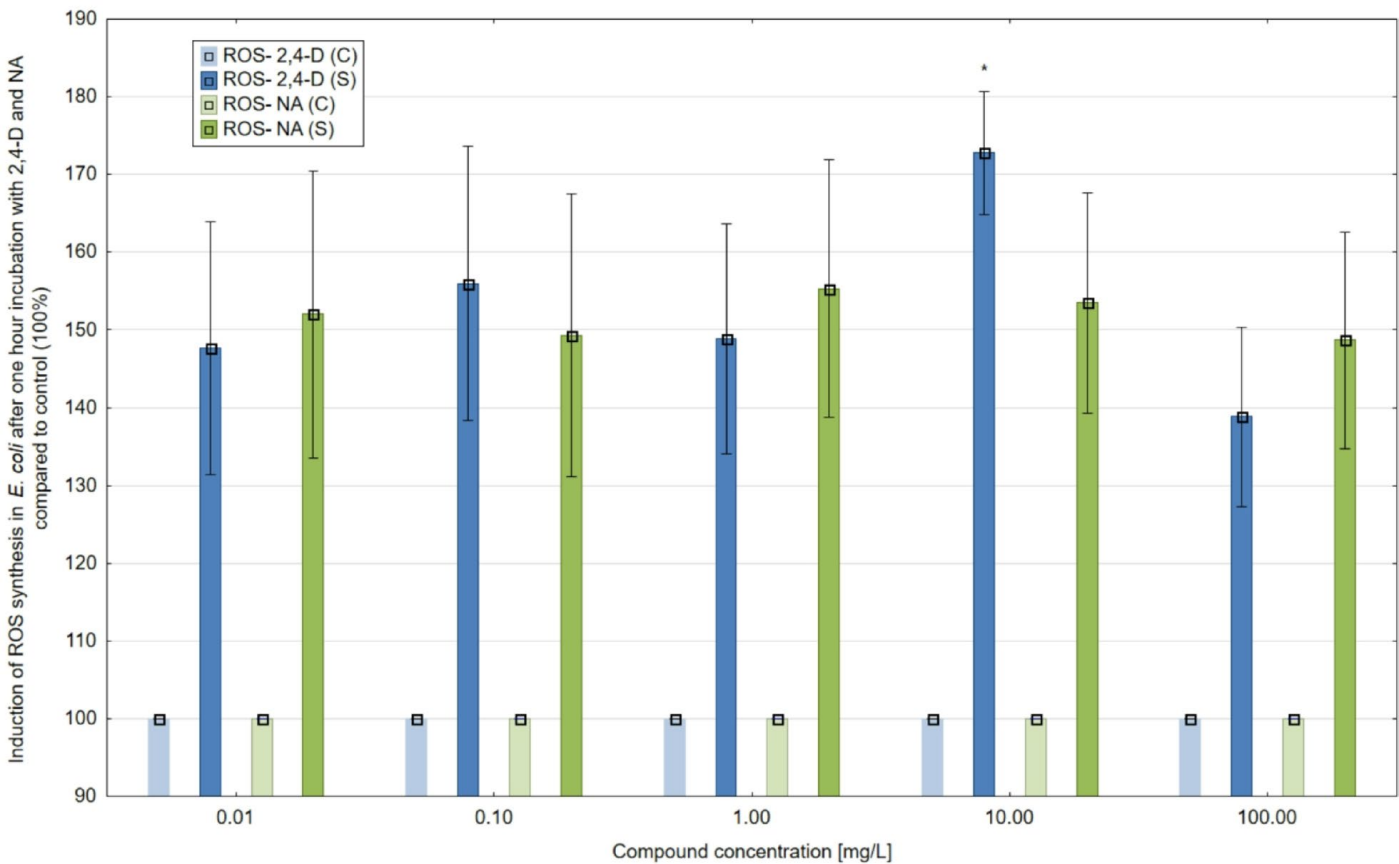
ROS synthesis (%) in *E. coli* ATCC-25922 after a one-hour exposure to 2,4-D and NA. Note: *- statistically significant difference at *p* = 0.05 between control samples. The results are presented as mean ± SD (standard deviation). Error bars indicate the SD of the mean of three independent replicates (*n* = 3).

Hourly exposure of *E. coli* culture to 2,4-D and NA at concentrations of 100, 10, 1, 0.1 and 0.01 mg/L intensively induced ROS synthesis compared to the control (100%) (Fig. 8). The strongest stimulation of ROS generation in *E. coli* to more than 170% was detected at a concentration of 10 mg/L and for 2,4-D. This herbicide increased ROS synthesis from almost 140% to over 155% at the other concentrations tested (100, 1, 0.1 and 0.01 mg/L). NA tested at a concentration of 1 mg/L induced ROS synthesis most strongly to over 155% in *E. coli* cultures. For the other NA concentrations tested (100, 10, 0.1 and 0.01 mg/L), the values for the increase in ROS generation in *E. coli* ranged from more than 148% to more than 153%. Comparing the pro-oxidant potential of 2,4-D with NA, it was found that the levels of induction of ROS synthesis in *E. coli* exposed to these compounds differed. The most significant differences between 2,4-D and NA were detected at 10 mg/L concentrations. Herbicide 2,4-D tested at 10 mg/L compared to an equivalent concentration of NA showed a stronger pro-oxidant effect and an almost 20% more intense synthesis of ROS.

In the results obtained, a concentration of 10 mg/L had maximum effects, leading to the highest levels of ROS synthesis (up to more than 170% compared to the control). It is likely that at this concentration, the 2,4-D acting on *E. coli* cells moves most intensively into the cell interior. In contrast, at the higher tested concentration of 2,4-D of 100 mg/L having a toxic effect on *E. coli* cells (as shown for a concentration of 100 mg/L in the 2,4-D antimicrobial activity assay), membrane permeability is impaired and respiratory chain enzymes are down-regulated, resulting in a decrease in ROS synthesis compared to a concentration of 10 mg/L. 2,4-D is detected in water at ng/L or µg/L concentrations. In contrast, in the study presented here, the most intense levels of oxidative stress (ROS synthesis) were detected at concentrations significantly higher than the concentrations of 2,4-D detected in the environment. Compared to real-world exposure scenarios, there are situations, e.g. agricultural runoff, when pesticides in higher concentrations enter waters and can affect living organisms by causing oxidative stress.

For ROS synthesis, only one statistically significant difference was observed, which occurred in the studies conducted on the effect of 2,4-D at a concentration of 10.00 mg/L. In the other samples, no statistically significant differences were observed between the control and studied samples, even though the ROS values for the individual study samples showed an apparent increase comparable to the control sample. For 2,4-D concentrations 100, 1, 0.1 and 0.01 mg/l, no statistically significant differences were detected (*p* = 0.05), which may suggest, the level of ROS synthesis in the cell is strictly dependent on the concentration of 2,4-D acting on the cell. Similarly, and for NA, no statistical significance was found in ROS synthesis between control and test samples.

The results obtained in this paper are specific only to the *E. coli* and *C. albicans* strains analysed. These results may not generalise to other microbes or eukaryotic cells.

## Discussion

This study determined the antimicrobial activity 2,4-D against *E. hormaechei* LBM ATCC-700323 and C. *albicans* ATCC-10231 strains in the 100, 10, 1, 0.1 and 0.01 mg/L concentration range. The results we obtained regarding the toxicity of 2,4-D are consistent with the results of experiments performed by previous researchers in which the antimicrobial activity of 2,4-D against bacteria and fungi was established^39^. While many studies focus on the effects of 2,4-D on soil microbial community structures and functions, there are fewer that extensively describe the antimicrobial activity of 2,4-D against microorganisms from laboratory collections.

Tyler (2022) reported a decrease in the growth of culturable bacteria in soils up to 15 days after 2,4-D high-dose spraying^40^. The toxic effect of this herbicide was detected in common soil bacteria such as *Azospirillum brasilense* and some *Rhizobium sp*. A completely different effect of 2,4-D was encountered in the case of soil fungi, the population size of which was stimulated under the influence of this herbicide’s high concentrations. A wide variety of effects 2,4-D on soil nutrient-cycling activities and the enzymatic activity of soil microorganisms were also found. In studies on nitrogenase activity in soil microorganisms, an inhibitory effect of 2,4-D on the activity of this enzyme was found in *Klebsiella pneumoniae* and *Nostoc muscorum*. In contrast, for *Enterobacter agglomerans*, an increase in the activity of this enzyme was observed. A similar pattern of 2,4-D reactivity was observed in soil nitrifiers, with autotrophic nitrifiers inhibited under 2,4-D and heterotrophic ones stimulated^40,41^.

The studies carried out in this work demonstrated the differential sensitivity of *E. hormaechei* and *C. albicans* to the same concentrations of 2,4-D. At the highest tested concentration of 100 mg/L, 2,4-D inhibited the growth of *C. albicans* more intensively compared to *E. hormaechei*. In contrast, lower concentrations of 2,4-D inhibited the growth of *E. hormaechei* cultures more intensively than *C. albicans*. In studies of the effects of pesticides on the activity of soil microorganisms, strains of both bacteria and fungi have so far been detected that exhibit the ability to biodegrade pesticides, including 2,4-D, and among these are *Sphingomonas*, *Pseudomonas*, *Cupriavidus*, *Achromobacter*, *Ochrobactrum*, *Mortierella*, and *Umbelopsis*. In addition, some enzymes and genes responsible for the biodegradation of 2,4-D have been identified and could be used to genetically engineer new microorganisms with the ability to biodegrade this herbicide^42^. Subsequent work by Itoh et al. (2013) described research conducted on a collection of fungi isolated from the soil. From this collection, 20 strains were selected that degraded 2,4-D, of which *Aspergillus penicilloides* and *Mortierella isabellina* strains demonstrated the highest herbicide degradation efficiency^36^.

The results of ROS synthesis in *E. coli* obtained in our experiments after exposure to 2,4-D at concentrations of 100, 10, 1, 0.1, and 0.01 mg/L indicate that oxidative stress is the mechanism of herbicide toxicity. 2,4-D at all concentrations tested induced an increase in ROS generation compared to the control. This herbicide most intensively induced ROS synthesis in bacterial cells at 10 mg/L (up to over 170%) and 0.1 mg/L (up to over 150%). Our results are consistent with the data from the work of previous authors Bukowska et al. (2016) and Sharifi Pasandi et al. (2017), in which a significant cytotoxic effect and oxidative stress generation, resulting in damage to DNA, proteins and lipids, in the tested human or animal cell lines such as cerebellar granule cells (CGC), breast cancer cell line, normal and cancerous lung cells, human peripheral blood mononuclear cells (PBMCs), human umbilical vein endothelial cells (HUVECs) and human gingival fibroblasts (HGF2) was demonstrated under the influence of 2,4-D^43,44^. The main mechanisms of 2,4-D toxicity have been identified as oxidative stress and destruction of cellular structures and important molecules such as DNA, RNA, proteins and lipids, depletion of antioxidants, and induction of apoptosis^44,45^. During the cellular defense response to oxidative stress conditions and disruption of cellular antioxidant activity, the HSF (Heat shock factor) activation and the organized networks of heat shock proteins (HSPs) biosynthesis may occur^46^. One of the main functions of HSPs is their chaperoning activities and cellular protein protection (proper protein folding and repair, degrading irreparable peptides) from the damaging effects of oxidative stress^47,48^. It is imperative to protect enzymes belonging to the antioxidant system^46^. Increased ROS levels in the cell can lead to DNA damage and gene deactivation (genotoxic effect). The results of the 2,4-D genotoxicity studies obtained in our work using the biosensor strain *E. coli recA: luxCDABE* indicate the genotoxic potential of 2,4-D. The highest levels of genotoxicity, up to almost 160%, were detected for 2,4-D after 2 and 24 h at the highest herbicide concentrations tested of 100 and 10 mg/L. Our findings agree with the results of Lerro et al. (2017) work in which the genotoxic potential of 2,4-D has been documented^49^. The authors of this study tested 225 urine samples from farmers in contact with 2,4-D during agricultural work for oxidative stress markers. In most of the samples tested, the authors detected elevated levels of 8-OHdG – a biomarker of DNA oxidation damage- and 8-isoPGF, a leading product of lipoprotein peroxidation. The results showed that the primary mechanism of the genotoxic effect of 2,4-D is oxidative DNA damage. They concluded that oxidative stress induced by exposure to 2,4-D can lead to the development of cancer and a range of chronic illnesses^42,49^.

Regarding 2,4-D exposure to 2,4-D has been linked with the induction of carcinogenesis, including prostate cancer^28,50–52^. As highlighted, 2,4-D can potentially induce oxidative stress by increasing ROS (reactive oxygen species) synthesis. Elevated ROS levels can cause DNA fragmentation, resulting in genotoxic effects, and damage RNA, proteins, and cellular lipids. This leads to lipid peroxidation, protein degradation, and the inactivation of essential cellular enzymes. Elevated ROS levels can alter the expression of transcription factors and suppressor genes; if these changes are not repaired by the cellular repair mechanisms, they can promote cancer proliferation, including prostate cancer^46,47^. Finally, oxidative stress affects miRNA activity, which can regulate the expression of many genes, including proto-oncogenes and tumour suppressor genes^28,42,53–58^.

In the studies presented, it was shown that 2,4-D has toxic and genotoxic effects and generates ROS synthesis and oxidative stress. Considering the huge amount of this herbicide, as it is used worldwide, 2,4-D poses a serious threat to human health. Elevated synthesis of ROS above physiological levels induces oxidative stress, which is the primary mechanism leading to the development of many cardiovascular diseases, diabetes and cancer. Enhanced ROS synthesis and oxidative DNA damage following exposure of cells to 2,4-D can lead to inhibition of the expression of cell-survival-essential genes such as p53 and genes of the organized networks of heat shock proteins (HSPs), leading to a significant decrease in the cell’s protective and repair potential and endocrine disruption. Following it, 2,4-D may affect numerous cells by changing the activity of heat shock proteins. Pesticides induce oxidative stress, leading to the dysfunction of cellular proteins, including HSPs, or impairment of their functions. Impairing their protective function towards the p53 protein may lead to partial or complete deactivation of the p53 protein^47,48^. Finally, without a well-functioning “guardian of the genome”, the complete dysregulation of genome stability, cell homeostasis and induction of cancer development may occur. That genotoxicity network can consequently lead to genome instability and cancer induction or cell death. In the cell, dysregulated levels of gene expression, or their inhibition, can lead to destabilised genome-wide activity, gene mutations and impaired cell signal transduction and cancer initiation. It’s like a ball that hits a goal, the whole goal shakes under its impact.

There is a link between the effects of pesticides on the expression levels of genes involved in estrogenic cell signal transduction pathways and the induction of breast cancer (BC). The main molecular mechanism of tumour induction is the mutagenic and non-mutagenic activity of pesticides as carcinogens. Another mechanism concerns the indirect activity of pesticides in the cell as biochemical modifiers and hormonal deregulators^50–53,61^.

The mechanisms mentioned above explain the involvement of 2,4-D in cancer development^47,53,59–62^. Studies have shown that GSH, as a major component of the human antioxidant system, has the potential to reduce the harmful effects of 2,4-D on the human body. We can maintain proper levels of GSH in our cells by leading a healthy lifestyle, eating foods rich in GSH and other antioxidants such as, vitamins A, C and E as well as selenium and polyphenols.

In our research, an in vitro experiment using the YES/YAS bioassay was conducted to detect the estrogenic and androgenic activities of 2,4-D as a potential EDC. Our findings indicated that 2,4-D did not exhibit any EDC activity in vitro. Considering that endocrine-disrupting chemicals (EDCs) can act at very low doses and exhibit non-monotonic dose-response relationships, we tested eight concentrations of 2,4-D (100, 10, 1, 0.1, 0.01, 0.001, 0.0001, and 0.00001 mg/L). The assessment of its estrogenic and androgenic activity showed no effect on hERα or hAR receptors at any of the tested concentrations. These results are consistent with findings by Orton et al. (2009)^63^Coady et al. (2014)^64^and Neal et al. (2017)^65^who also reported no evidence of interaction between 2,4-D and estrogen or androgen receptors in vitro. Moreover, a lack of in vivo endocrine effects in animal studies, including fish, amphibians and rats, has been reported by Coady et al. (2013)^66^ and Marty et al. (2013)^67^. The lack of estrogenic and androgenic 2,4-D activity observed in our study should be interpreted carefully, as in vitro models do not fully replicate the complexity of in vivo conditions. In vivo xenobiotics like 2,4-D can undergo biotransformation in the liver, resulting in metabolites with different biological activities, including potentially endocrine-disrupting ones, not detected in the original compound during YES/YAS testing^81^. Therefore, the negative results observed in the YES/YAS assay for 2,4-D do not definitively exclude endocrine potential in vivo. Nonetheless, our results indicate that the crucial mechanism of 2,4-D cancerogenesis is the induction of ROS, which can activate a number of oncogenes.

The results of our study demonstrated that, in the tested mixtures of 2,4-D and NA with GSH, depending on the herbicide and antibiotic concentration used, the addition of GSH has a bidirectional effect, reducing the toxicity and genotoxicity of 2,4-D and NA and enhancing the values of these parameters. GSH, as a non-enzymatic antioxidant, plays a protective role against free radicals and pro-oxidants and as a cofactor for antioxidant and detoxification enzymes such as glutathione peroxidases, glutathione S-transferases, and glyoxalases^68^. Glutathione is a known regulator of cellular redox homeostasis^69^. The antioxidant action of glutathione is associated with detoxifying hydrogen peroxide, organic peroxides and other reactive oxygen species, exo- and endogenous electrophilic compounds, and its ability to chelate dangerous metal ions. Glutathione is also involved in restoring damaged cell components, mainly proteins and lipids of cell membranes and DNA. In addition, the compound maintains the standard redox potential of cells, which is essential in regulating intracellular metabolism, cell growth and differentiation processes and apoptosis^68,70^. The toxicity- and genotoxicity-reducing effects of GSH in mixtures with 2,4-D obtained in our experiments can be explained based on the above-mentioned molecular mechanisms of action of GSH as one of the most important cellular antioxidants.

In mixtures containing 2,4-D at a concentration of 0.01 mg/L with GSH, a slight increase in 2,4-D toxicity (by approximately 6%) was observed when GSH was added compared to the application of 2,4-D alone. A similar effect of GSH was detected in a mixture with NA applied at a concentration of 1 mg/L, where the addition of GSH induced an enhancement of more than 8% in the genotoxicity of NA compared to NA applied alone. According to the scientific literature, GSH’s paradoxical effects (toxicity enhancement) are explained that, under enabling conditions, the reduced form of GSH (GSSG) acts as a pro-oxidant. Moreover, Rebrin and Sohan (2008) showed that exposure of cells to toxic chemicals (e.g. ethanol) leads to a depletion of mitochondrial GSH^71^. In living cells, the primary determinant of the cellular redox state is the GSH/GSSG ratio and the efficiency of *de novo* GSH biosynthesis. First, 2,4-D may affect the activity of enzymes belonging to the cellular oxidative system, such as selenium-dependent glutathione peroxidase (Se-GSH-px), glutathione reductase (GSSG-rx) and glutathione transferase (GST). Secondly, 2,4-D showing pro-oxidant activity in living cells can interfere with the proper functioning of enzymes responsible for optimal regulation of GSH biosynthesis, GSH/GSSG ratio and cellular redox homeostasis, which may lead to a significant weakening of the cell’s antioxidant potential and a deepening of the toxic and genotoxic effects of 2,4-D^69,72^.

It is known that GSH has a “Janus-faced” role, meaning it exhibits both beneficial and detrimental effects depending on the context. In healthy, normally functioning cells, GSH is an antioxidant and its concentration depends on many parameters, including cellular oxygenation, extracellular pH and redox balance. In cells under oxidative stress and in cancer cells, redox balance, cellular hypoxia and pH changes occur. Moreover, in cancer cells, elevated GSH levels can contribute to drug resistance and tumour progression^80^. In the light of the above, the toxic effects of 2,4-D on the cell can induce shifts in the redox balance, which disrupts normal GSH synthesis and activity, leading to pro-oxidative effects of GSH in the mixtures with low concentrations of 2,4-D. In our experiments, the obtained pro-oxidative activity of GSH can support the hypothesized Janus-faced character of the redox axis.

## Conclusions

This study determined the toxicity and genotoxicity of 2,4-D, a widely used herbicide, and its mixtures with GSH on various microorganisms. Results showed significant antimicrobial and genotoxic activity of 2,4-D towards *E. homaechei* and *C. albicans*. Based on the analysis of ROS synthesis levels in *E. coli*, we also detected that oxidative stress is the mechanism of 2,4-D toxicity and genotoxicity. Considering the literature data and the results obtained in this work, which indicate the potential for toxic effects on living organisms, including the impact of possible cancer induction, 2,4-D can be classified as an environmental micropollutant dangerous to humans. Consequently, the use of this herbicide must be approached very rationally. Above all, developing more effective methods for removing 2,4-D from wastewater and surface waters as potential drinking water sources is crucial. To protect our bodies from the harmful effects of 2,4-D, we can follow a diet rich in natural antioxidants, including GSH. This is confirmed by the results we obtained and described in this paper. In our results, in mixtures with 2,4-D, depending on the concentration of herbicide used, the addition of GSH at a concentration of 1 mg/L significantly modulates the toxicity and genotoxicity of the herbicide, leading to the reduction of the values for these parameters. It indicates that GSH taken with food or supplements can reduce the adverse effects of 2,4-D in living cells and protect cells from cancer induction. From the point of view of cellular oxidative protection and cancer prevention, it is also essential to provide the body with other active antioxidants, such as vitamins C, A, E, D and selenium, among others.

## Materials and methods

### Chemical compounds and analysis

2,4-D, GSH and nalidixic acid (NA) were purchased from Sigma-Aldrich (Sigma-Aldrich, UK) and used without further purification. All the chemical compounds tested were dissolved in dimethyl sulfoxide (DMSO). To reduce the effect of DMSO on living cells, its solutions were diluted in 0.86% NaCl in a ratio of 1:9 (100 µL DMSO and 900 µL 0.86% NaCl). It is known that DMSO affects gene expression levels and cell metabolism, so 10% DMSO, which is non-toxic to cells was used. The standard used in the study was NA. 2,4-D and NA were tested at 0.01, 0.1, 1, 10 and 100 mg/L concentrations. In contrast, GSH and NA in mixtures with all concentrations of 2,4-D and GSH in mixtures with NA were tested at 1 mg/L. Concentrations of 2,4-D were selected for the experiments that were within the range of the maximum concentration of this herbicide (50 µg/L) set by the United States Environmental Protection Agency (USEPA) for drinking water (USEPA, Cai et al., 2023). Moreover, concentrations (0.01 mg/L) corresponding to the concentrations of pesticides detected in surface waters were selected for analysis. In contrast, higher concentrations of the pesticide, up to 100 mg/L, were used to investigate whether the molecular mechanism of toxicity of 2,4-D is concentration-dependent. Then, in the mixtures, the concentration of 1 mg/L of GSH was used. According to the scientific literature, the concentration of GSH in cells depends on their type and ranges from 5 to 10 mM, while the level of GSH in the serum is much lower (20 µM)^29,31–35^. In experiment, a lower concentration than physiologically was used – 1 mg/L. This is the concentration that was selected experimentally. For higher (10 mg/L) as well as lower concentrations (0.1; 0.01 and 0.001 mg/L), we obtained unsatisfactory results. Analyses of a given parameter were performed in at least three independent series with at least 3 repetitions of each result (*n* = 3) in a single series.

## 2,4-D and its mixtures with GSH and NA antimicrobial activity

### The microorganisms used

The antimicrobial activity of 2,4-D, NA, GSH and their mixtures with NA and GSH was estimated using *E. homaechei* LBM ATCC-700323 and *C. albicans* ATCC-10231, representing bacteria and fungi. The microorganisms were purchased from ATCC (American Type Culture Collection, USA). The two strains *E. hormaechei* and *C. albicans* were selected as a model for Gram-negative bacteria and opportunistic fungi.

### The Estimation of the antimicrobial activity of chemicals tested and their mixtures

The antimicrobial activity of chemicals tested and their mixtures was estimated based on the growth inhibition effect of *E. homaechei* and *C. albicans* cultures. The microorganisms were cultured overnight at 37 °C on Luria Bertani broth (LB) (casein peptone 10 g/L; yeast extract 5 g/L and NaCl 10 g/L, pH 7.0). Then, the cultures were diluted with fresh LB broth and incubated in a shaking water bath (130 rpm) at 37 °C to the logarithmic growth phase (OD_600_ = 0.2). After that, the samples were prepared in 96-well plates by adding 100 µl of microorganism cultures to the 100 µl of appropriate concentrations of 2,4-D, NA, GSH and their mixtures with NA and GSH. The samples were mixed and incubated at 37 °C for 24 and 48 h. The control sample did not contain any chemicals. The growth inhibition effect of the tested microorganism culture after 2,4-D, NA, GSH and their mixtures with NA and GSH treatment was determined based on Optical Density (OD) values, measured at 600 nm with the use of a GloMax^®^ microplate reader (Promega, MA, USA). The obtained results were presented as a percentage of growth inhibition/stimulation of the tested microorganism culture compared to the control sample. Experiments were done in triplicate.

### The Estimation of the antimicrobial activity of chemicals tested and their mixtures

To date, some research papers have used an *E. coli* microbial biosensor strain with a plasmid-based transcriptional fusion of the genotoxin-sensitive *recA* promoter with the *lux* gene as a reporter to determine the genotoxicity of drugs and their metabolites, pesticides, metals, polychlorinated biphenyls (PCBs), organic pollutants, chromium, arsenic, lead and petroleum hydrocarbons in surface water, wastewaters and soils^38,73^. The genotoxin-sensitive *recA* promoter is part of the stress response (SOS) pathway, consisting of an inducible network of genes involved in DNA repair^74,75^. For genotoxicity assessment of 2,4-D, NA, GSH and their mixtures with NA and GSH, the *E. coli* RFM443 *recA:luxCDABE* bacterial biosensor with plasmid construct containing genotoxin inducible-*recA* promoter transcriptionally fused to the *luxCDABE* reporter gene was used. The chemicals and their mixtures were applied at 0.01, 0.1, 1, 10 and 100 mg/L concentrations. In this assay, the genotoxic potential of the test chemicals and their mixtures is directly proportional to the level of induction of the *recA* promoter and luminescence emission. To obtain *E. coli recA:luxCDABE* cells in the logarithmic growth phase, a 24 h culture of the bacterial strain, conducted at 37 °C on LB broth, was refreshed by dilution with LB broth and placed for 2.5 h (OD_600_ = 0.2) in a shaking (130 rpm) water bath at 37 °C. On a 96-well white plate, the appropriate solutions of 2,4-D, NA, GSH and their mixtures with NA and GSH were added to the LB broth cultures of *E. coli recA:luxCDABE* and incubated for 2 and 24 h at 37 °C. To standardize the luminescence response of the *E. coli recA:luxCDABE* culture for all test samples and the control samples, the OD at 600 nm was spectrophotometrically determined. Luminescence was measured (using a GloMax^®^ microplate reader, Promega, MA, USA) for all tested and control samples and according to the method described by Melamed et al. (2012)^76^. To account for cell density variations the luminescence data were normalised according to the formula: L_f_=L/OD_600_, where: L_f_ – final luminescence; L– the luminescence values read from spectroluminometer; OD_600_ – the optical density of *E. coli* culture, that showed the bacteria concentration. The results were read after 2 and 24 h. The results are presented as the percentage (%) of induction/inhibition of *recA* promoter and luminescence compared to the control sample. The experiment was performed in triplicate.

### Yeast Estrogen screen (YES) and androgen screen (YAS) bioassays for 2,4-D

A solution of 2,4-D was prepared in ethyl acetate to achieve a final concentration of 100 µg/mL. A volume of 150 µL of this solution was dispensed into a 96-well polypropylene plate. Serial dilutions were performed in a 1:3 ratio using ethyl acetate. E2 and DHT were used as positive controls for the YES and YAS bioassay, respectively, with ethyl acetate serving as the blank control. The assay detects estrogenic activity at ≥ 4.2 × 10⁻¹² M for 17β-estradiol and androgenic activity at ≥ 2.1 × 10⁻¹⁰ M for dihydrotestosterone (limit of detection for reference compounds). The tested concentrations of 2,4-D were 0.00001, 0.0001, 0.001, 0.01, 0.1, 1, 10, and 100 mg/L. The samples were left to evaporate at room temperature overnight. For the YES and YAS assays, *Saccharomyces cerevisiae* strain (Tigret Poland) was cultured in YPG medium (BLT, Poland) containing streptomycin (0.5 mg/mL) and ampicillin (0.5 mg/mL). The yeast was incubated overnight at 28°C. After incubation, the culture was centrifuged at 10,000 × g for 10 min, and the cells were resuspended in fresh medium to reach an OD_690_ of 0.4. Endocrine activity was measured following the protocol of Średnicka et al. (2024)^77^. Evaporated test samples were reconstituted with 120 µL of 1% DMSO and mixed with 60 µL of the yeast suspension. The mixtures were incubated overnight at 28°C in a humidified plastic chamber. After incubation, growth was monitored by measuring optical density at 690 nm. To terminate the cultures, 20 µL of 0.1% Triton X-100 and 30 µL of lyticase (1 mg/mL in 0.1 M phosphate buffer, pH 7.5, containing 50 mM mercaptoethanol) were added, and the plates were incubated for 1 h at 35°C. Subsequently, 50 µL of chlorophenol red-β-D-galactopyranoside (CPRG) solution (1.0 mg/mL in phosphate buffer, pH 7.5) was added. CPRG hydrolysis was measured at 570 nm after 15 min of incubation at 35°C in the dark using the Gen5™ software on a Biotek Synergy H1 plate reader (Gen5™ software Data Acquisition Module, SoftMax^®^ Pro, version 1. 3. 0, URL link: https://help.bioassay.de/framework/topics/biotek_gen5_dam.html). Agonist activities targeting hERα and hAR receptors were determined based on the collected data, and dose-response curves were plotted to illustrate the results.

### Oxidative stress determination

To test whether oxidative stress could be a potential mechanism of 2,4-D toxicity, the level of ROS synthesis was determined in the *E. coli* ATCC-25922 strain incubated with this herbicide.

### ROS generation

Oxidative stress (OS) is a condition in which an imbalance occurs in the synthesis of ROS and the activity of the antioxidant system in a living cell. OS causes damage to cellular macromolecules, DNA, RNA, proteins and lipids and ultimately contributes to cellular dysfunction and death. Also, many environmental factors, such as radiation and chemicals, including pesticides, can generate excessive ROS and oxidative stress^78,79^.

In this study, the level of intracellular ROS generated by *E. coli* ATCC-25922 strain exposition to 2,4-D was presented. Nalidixic acid was used as a standard, whereas 7′-dichlorofluoresceindiacetate (DCFH-DA) (Sigma-Aldrich, UK) was applied to assess the potential for ROS synthesis induction in *E. coli* treated with 2,4-D at concentrations of 0.01, 0.1, 1, 10 and 100 mg/L, according to the method described in our earlier works^38^. After the overnight incubation of *the E. coli* strain at 37°C in LB broth, the culture was refreshed with LB broth, and the bacterial culture was grown in a water bath at 37°C with shaking (130 rpm) to reach the log phase (OD_600_ = 0.2). Afterwards, appropriate concentrations of 2,4-D and DCFH-DA at a final concentration of 5 µM were added to the *E. coli* culture and incubated at 37°C for 1 h. No 2,4-D was added to the control sample. Next, the DCF fluorescence intensity was measured using a GloMax^®^ microplate reader (Promega, MA, USA) at the excitation wavelength of 485 nm and the emission wavelength of 535 nm. In this method, the level of ROS synthesis in *E. coli* cells is directly proportional to the 2,4-D-treated samples’ fluorescence intensity. Simultaneously with the fluorescence intensity measurements for all samples, including the control, the optical density (OD) value measured at 600 nm was monitored. The obtained results were presented as a percent (%) of ROS synthesis increase compared to the non-treated control culture (100%). The experiment was repeated three times.

### Statistical analysis

The results of the laboratory studies were subjected to statistical analysis using the Tukey test for equilateral samples. The results of the statistical analysis were verified for changes in individual indicator values (growth inhibition, induction of promoter *recA* and ROS synthesis) for the compounds under consideration and their mixtures at concentrations of 0.01, 0.10, 1, 10, 100 mg/L and incubation times resulting from the conditions described in the analytical methodology. The level of statistical significance was taken as α = 0.05. In addition, before selecting a statistical test to verify the occurrence of the least significant differences, the data set was subjected to the Bartlett test to verify homogeneity of variance and the Shapiro-Wilk test to determine the normality of the distribution of the variables adopted for analysis.

## Data Availability

The datasets used and analysed during the current study are available from the corresponding author on reasonable request. All data generated or analysed during this study are included in this published article.
